# Elevated circulating cell-free mitochondrial DNA in fabry disease: insights into inflammatory activation

**DOI:** 10.3389/fimmu.2025.1706045

**Published:** 2026-01-07

**Authors:** Yujing Yuan, Xinyu Zhang, Yawen Zhao, Yikang Wang, Jingyi Huang, Qingqing Wang, Xueqin Song, Jianwen Deng, Zhaoxia Wang, Yun Yuan, Wei Zhang

**Affiliations:** 1 Department of Neurology, Peking University First Hospital, Beijing, China; 2 Department of Neurology, The First Affiliated Hospital of Zhengzhou University, Zhengzhou, China; 3 Department of Neurology, The Second Hospital of Hebei Medical University, Shijiazhuang, Hebei, China; 4 Key Laboratory of Clinical Neurology, Ministry of Education, Hebei Medical University, Shijiazhuang, Hebei, China; 5 Neurological Laboratory of Hebei Province, Shijiazhuang, Hebei, China; 6 Beijing Key Laboratory of Neurovascular Diseases, Beijing, China; 7 Rare Disease Medical Center, Peking University First Hospital, Beijing, China

**Keywords:** ccf-mtDNA, enzyme replacement therapy, Fabry disease, inflammatory cytokines, mitochondrial dysfunction

## Abstract

**Objective:**

This study aimed to investigate mitochondrial dysfunction and its role in the pathogenesis of Fabry disease (FD) by analyzing circulating cell-free DNA (ccf-DNA) in patients with FD.

**Methods:**

Sixty-six FD patients and 21 healthy controls (ctrls) were enrolled. Levels of plasma mitochondrial- (ccf-mtDNA) and nuclear-derived ccf-DNA (ccf-nDNA) were quantified by quantitative reverse-transcription PCR (RT-qPCR), and 14 inflammatory cytokines were measured in treatment-naïve patients. Associations among ccf-DNA levels, cytokine profiles, disease biomarkers, and clinical markers were analyzed, with subgroup analyses stratified by sex, genotype, clinical subtype, and disease severity.

**Results:**

Treatment-naïve patients exhibited significantly higher ccf-mtDNA (*z*=–4.530, *P*-adj<0.001) and mtDNA/nDNA ratio (*z*=–2.613, *P*-adj=0.014) compared with ctrls. In the long-term enzyme replacement therapy (ERT) group (> 12 months), ccf-mtDNA copy number remained elevated (*z*=–3.141, *P*-adj=0.006), whereas the mtDNA/nDNA ratio did not differ significantly (*z*=–1.013, *P*-adj=0.311). No differences in ccf-nDNA were observed between treatment-naïve patients or the long-term ERT group compared with ctrls. Receiver operating characteristic analysis demonstrated the strong diagnostic performance of ccf-mtDNA (area under the curve=0.860), with 70% sensitivity and 91% specificity at an optimal cut-off value of 1,793,188.04 copies. Both ccf-mtDNA and mtDNA/nDNA ratio correlated positively with inflammatory cytokines including interleukin-17F and tumor necrosis factor-β, with stronger associations observed in male patients with classic FD. No correlations were observed with disease duration, α-galactosidase A activity, plasma globotriaosylsphingosine or clinical markers after adjustment for age and sex. Similarly, ccf-DNA measures did not differ significantly by sex, *GLA* mutation type (truncated *vs*. non-truncated), FD subtype (classic *vs*. non-classic), or across subgroups defined by disease severity or organ involvement (high *vs*. low MSSI, with or without hypertrophic cardiomyopathy, with or without chronic kidney disease, mild *vs*. severe white matter lesions, with or without neuralgia, or mild *vs*. severe pain).

**Conclusions:**

Mitochondrial dysfunction, reflected by elevated ccf-mtDNA, is implicated in FD pathogenesis and may be linked to inflammatory activation. ccf-mtDNA represents a promising diagnostic biomarker for FD, potentially offering an additional therapeutic target when combined with ERT.

## 1 Introduction

Fabry disease (FD; OMIM #301500), also known as Anderson-Fabry disease, is a rare X-linked lysosomal storage disorder caused by mutations in the *GLA* gene encoding α-galactosidase A (α-GalA), which is located on chromosome Xq22.1. These mutations result in reduced or absent α-GalA activity, leading to lysosomal accumulation of globotriaosylceramide (Gb3) and its deacylated form, globotriaosylsphingosine (lyso-Gb3) in multiple tissues, ultimately causing cellular dysfunction and multi-organ damage (1). Clinically, FD is classified into classic and non-classic phenotypes. Classic FD is characterized by little or no residual α-GalA activity and widespread multi-system involvement; male patients with the classic phenotype are often used as representative cohorts for evaluating new biomarkers (2–4). In contrast, non-classic FD retains partial α-GalA activity, with organ damage typically limited to specific systems, most commonly the heart or kidneys (5). Enzyme replacement therapy (ERT) remains the cornerstone of FD treatment.

Circulating cell-free DNA (ccf-DNA) has recently been recognized as a damage-associated molecular pattern, including mitochondrial-derived ccf-DNA (ccf-mtDNA), a marker of mitochondrial dysfunction, and nuclear-derived ccf-DNA (ccf-nDNA) (6). Elevated ccf-DNA levels have been reported in several disease states, underscoring their potential as biomarkers of disease activity and therapeutic response (7–10). Ccf-mtDNA release can occur through several mechanisms, including​ phagocytic clearance of damaged mitochondria. Once in the extracellular space, it can promote​ inflammatory responses through several pathways, including activation of the cyclic GMP–AMP synthase (cGAS)–stimulator of interferon genes (STING) pathway (10).

Both mitochondrial dysfunction and inflammatory signaling are central to FD pathogenesis (11, 12), although the underlying cascade networks are complex. Our previous work demonstrated upregulation of inflammatory cytokines in FD and their correlation with disease phenotypes (11). Additionally, ERT has been shown to partially attenuate FD-associated inflammatory activation. We hypothesized that ccf-DNA contributes to disease heterogeneity and inflammation in FD. This study aimed to assess ccf-DNA as a diagnostic biomarker of FD, and to investigate its potential role in inflammatory activation.

## 2 Materials and methods

### 2.1 Study population, clinical data collection, and assessments

**Figure 1:**
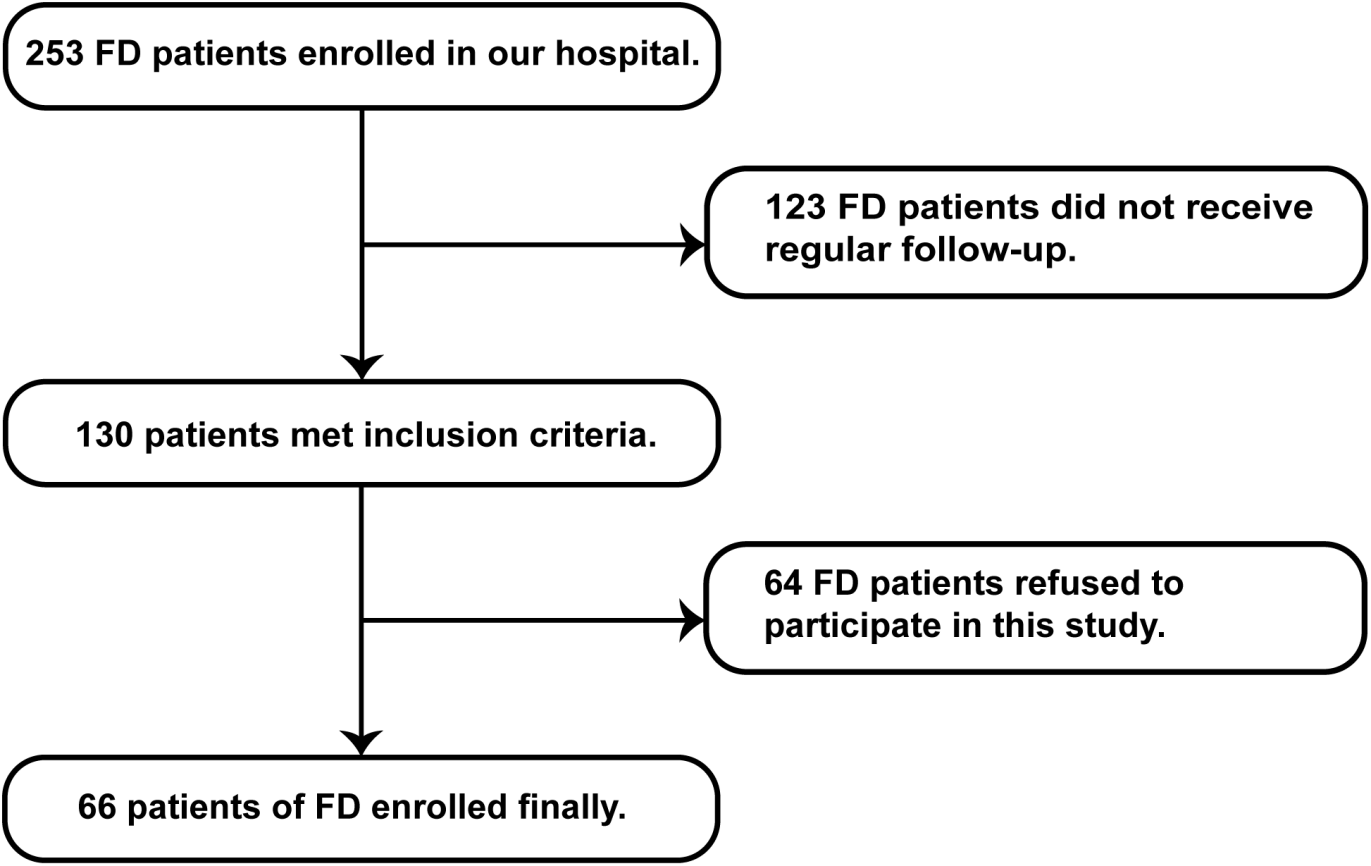
Flowchart of patient enrollment.

Patients with FD who received regular follow-up at the Department of Neurology, Peking University First Hospital, from September 2019 to March 2025 were recruited. Diagnosis was based on clinical history and laboratory findings (*GLA* gene mutation, α-GalA activity, and Lyso-Gb3 levels) according to the Chinese consensus on FD (13). Patients were excluded if they had serious comorbidities unrelated to FD, including significant liver or kidney dysfunction, blood glucose or lipid abnormalities, cardiovascular or cerebrovascular diseases, infectious diseases, autoimmune disorders, tumors, or other chronic illnesses, as determined by medical history and laboratory tests. Individuals with excessive alcohol consumption, illicit drug use, or heavy smoking were also excluded. Patients who declined to participate were not included (**Figure 1**). Age- and sex-matched healthy controls (ctrls) were recruited from the Health Management Center of Peking University First Hospital.

Comprehensive demographic and clinical data were collected for all patients. Multisystem involvement, including cardiac, renal, cerebral, and peripheral nervous system manifestations, was assessed using standardized clinical examinations and validated scales. Overall disease severity was evaluated using the Mainz Severity Score Index (MSSI) (11), cardiac involvement was evaluated using the left ventricular mass index (LVMI) (14, 15), and renal function was evaluated using estimated glomerular filtration rate (eGFR), urine albumin-to-creatinine ratio (ACR), urine protein-to-creatinine ratio (PCR), and 24-hour proteinuria (24h-P). Cerebral involvement was assessed through white matter lesions (WML) graded on the Fazekas scale using T2-FLAIR magnetic resonance imaging (16, 17), while peripheral nerve pain was assessed using the Visual Analogue Scale (VAS) for peak pain intensity over the past 24 hours, with scores ranging from 0 (no pain) to 10 (worst pain imaginable).

### 2.2 Study design

To investigate ccf-DNA expression status in FD, we compared ccf-mtDNA copy number, ccf-nDNA copy number, and mtDNA/nDNA ratio between treatment-naïve patients with FD and ctrls. To evaluate the impact of ERT, these parameters were compared between short-term ERT (≤ 12 months) group and ctrls, between long-term ERT (> 12 months (18)) group and ctrls, as well as between short-term and long-term ERT groups. To investigate associations with inflammation, we analyzed correlations between ccf-mtDNA, ccf-nDNA, mtDNA/nDNA ratio, and 14 inflammatory cytokines.

To evaluate the clinical value of ccf-DNA, we evaluated relationships between ccf-DNA measures and disease phenotype, genotype, and clinical markers. Correlations with disease duration, α-GalA activity, Lyso-Gb3, and clinical markers, including MSSI, LVMI, eGFR, ACR, PCR, 24h-P, Fazekas score, and VAS score, were examined after adjusting for sex and age. Mutations in *GLA* were classified as truncated or non-truncated (19). Non-truncated mutations included missense mutations, insertion and deletion variants (indels), and in-frame indels. Truncated mutations included splicing mutations, exon-intron boundary mutations, deep intronic indels, nonsense mutations, frameshift mutations, heterozygous deletions, and duplications. Subgroup analyses were performed by sex, disease subtype (classic vs. non-classic (5)), *GLA* genotype (truncated vs. non-truncated), as well as clinical features including low (< 20) *vs*. high (≥ 20) MSSI (11), hypertrophic cardiomyopathy (HCM; LVMI ≥ 88 g/m² in females and ≥ 102 g/m² in males) *vs*. non-HCM (14, 15), chronic kidney disease (CKD; eGFR<60 mL/min/1.73 m^2^ or the presence of proteinuria) *vs*. non-CKD (20, 21), mild (Fazekas <2) *vs*. severe (Fazekas ≥ 2) WML (16, 17), neuralgia *vs*. non-neuralgia, and mild (VAS<3) *vs*. severe (VAS ≥ 3) pain.

### 2.3 Laboratory assays

#### 2.3.1 Quantification of ccf-mtDNA and ccf-nDNA

Plasma ccf-mtDNA and ccf-nDNA copy numbers were quantified in patients with FD and ctrls. Plasma samples were processed promptly after blood withdrawal, undergoing repeated centrifugation to prevent cell lysis and eliminate potential cell contaminants. Ccf-DNA was extracted from 200 μL of plasma using the Plasma Free DNA Extraction Kit with magnetic beads (Tiangen, DP709).

Quantitative reverse-transcription PCR (RT-qPCR) was performed as previously described (22), targeting the mitochondrial NADH dehydrogenase 1 (*MT-ND1*) gene for ccf-mtDNA and the nuclear β-actin (*ACTB*) gene for ccf-nDNA. The 20 μL reaction mixture contained 2 μL of ccf-DNA, 1× UltraSYBR Mixture (CW2601), 0.2 μM of either *MT-ND1* or *ACTB* primers depending on template type, and nuclease-free water. Amplifications were performed in an AriaMx thermal cycler under the following conditions: 95°C for 10 min; 40 cycles of 95°C for 15 s and 60°C for 1 min; followed by 95°C for 30 s, 65°C for 30 s, and 95°C for 30 s. Data were analyzed using Agilent Aria version 1.71. *ACTB* served as the internal reference gene. Absolute copy numbers were determined according to a previously described method (23), in which cloned vectors containing *MT-ND1* and *ACTB* were serially diluted to generate linear standard curves (9, 10). The primers used were MT-ND1 forward (5´-CCACCTCTAGCCTAGCCGTTT A-3´), MT-ND1 reverse (5´-GGGTCATGATGGCAGGAGTAA T-3´), ACTB forward (5´-CTCCATCCTGGCCTCGCTGT -3´), and ACTB reverse (5´-GCTGTCACCTTCACCGTTCC-3´).

#### 2.3.2 Inflammatory cytokine assay

Peripheral blood was collected in ethylenediaminetetraacetic acid tubes from treatment-naïve patients with FD, and immediately centrifuged at 1,240 ×g for 5 min at 4°C. Plasma was isolated and frozen at -80°C before further processing. Samples were prepared and 14 inflammatory cytokines (interferon [IFN]-γ, interleukin [IL]-1β, IL-2, IL-4, IL-5, IL-6, IL-8, IL-10, IL-12p70, IL-17A, IL-17F, IL-22, tumor necrosis factor [TNF]-α, and TNF-β) were measured using a sandwich enzyme-linked immunosorbent assay kit (914002, QuantoBio, Tianjin, China) following the manufacturer’s instructions. Cytokine concentrations in each sample were detected using a flow cytometer (BeamCyte-1026M, Jiangsu, China) and analyzed using CYTOSYS 2.0 (Changzhou Bidako Biotechnology Co., Ltd., Changzhou, China) (11).

#### 2.3.3 Genetic analysis of the GLA gene

For probands with clinical suspicion of FD, we performed targeted next-generation sequencing of the GLA gene using dried blood spot samples, with simultaneous measurement of α-Gal A activity and lyso-Gb3 levels. All identified variants were confirmed by Sanger sequencing and segregation analysis within the family. For family members of confirmed FD patients, the known GLA variant was screened for by Sanger sequencing. Patients presenting with unexplained symptoms such as acroparesthesia, early-onset stroke, renal insufficiency, or hypertrophic cardiomyopathy were investigated using either whole-exome sequencing or targeted next-generation sequencing panels. Finally, for patients with clinical, biochemical, or pathological findings suggestive of FD but without an identified pathogenic variant in the GLA coding region after the above testing, long-read sequencing was employed to detect potential deep intronic or structural variants. All variants identified through strategies 3 and 4 were subsequently validated by Sanger sequencing.

### 2.4 Statistical analysis

Statistical analyses were performed with SPSS version 31.0 (SPSS, IBM, Chicago, IL). The Shapiro-Wilk test was used to assess variable distributions. The Chi-square (χ^2^) test was used to compare ratios. Normally distributed data are presented as the mean ± standard deviation and were compared using the independent samples t-test (t). Non-normally distributed data are presented as the median (interquartile range, IQR) and were compared using the Mann-Whitney U test (z). Associations between ccf-DNA (ccf-mtDNA, ccf-nDNA, and mtDNA/nDNA ratio) and clinical markers (MSSI, LVMI, eGFR, ACR, PCR, 24h-P, Fazekas score, and VAS score), as well as with inflammatory cytokines (IL-1β, IL-2, IL-4, IL-5, IL-6, IL-8, IL-10, IL-12p70, IL-17A, IL-17F, IL-22, TNF-α, and TNF-β), were assessed using Pearson’s *r*, with all results reported as Pearson’s *r* and *p* values. Partial correlations were computed to assess the relationships between ccf-DNA and inflammatory cytokines as well as clinical markers, adjusting for sex and age. Receiver operating characteristic curve analyses were conducted to distinguish between treatment-naïve patients with FD and ctrls, calculating the area under the curve (AUC), sensitivity, and specificity. The optimal cut-off points for each comparison were determined by maximizing Youden’s index (sensitivity + specificity – 1). An AUC > 0.5 with *p*<0.05 was considered indicative of diagnostic value. The Benjamini-Hochberg false discovery rate method was applied to control for type 1 error, and the *p* values of multiple comparisons were adjusted (*P*-adj). Two-tailed tests were used throughout, and statistical significance was set as *p* or *P*-adj<0.05.

## 3 Results

### 3.1 Cohort characteristics

This longitudinal cohort study included 66 patients with FD (35 males and 31 females) from 50 unrelated families, along with 21 ctrls (13 males and eight females) (**Table 1**). The median (IQR) age at sample collection was 38.50 (30.50, 50.00) years for patients with FD and 37.00 (30.00, 43.50) years for ctrls. Of the 66 patients with FD, two were asymptomatic but carried pathogenic *GLA* mutations with a positive family history; the remaining 64 patients had a median (IQR) age at symptom onset of 8.50 (7.00, 12.75) years and a mean disease duration of 25.70 ± 15.82 years. Across the 50 families, *GLA* sequencing identified 27 missense, 12 nonsense, three deletion, three splice-site, one indel, one duplication, one in-frame shift, one frameshift, and one intronic insertion mutations.

**Table 1:** Baseline demographic and clinical characteristics of patients with FD.

Variables	Patients with FD (n = 66)
Sex (male/female)	35/31
Onset age, Median (IQR)	8.50 (7.00,12.75)
Duration (mean ± SD)	25.70 ± 15.82
Classic phenotype, % (n)	59.09 (39)
Neuropathic pain, % (n)	83.33 (55)
Hypohidrosis, % (n)	60.61 (40)
Gastrointestinal discomfort, % (n)	46.97 (31)
Tinnitus, % (n)	56.06 (37)
Hearing loss, % (n)	37.88 (25)
Angiokeratoma, % (n)	46.97 (31)
Cornea verticillate, % (n)	42.42 (28)
Hypertension, % (n)	25.76 (17)
HCM, % (n)	53.03 (35)
Proteinuria, % (n)	51.52 (34)
Renal insufficiency, % (n)	39.39 (26)
Ischemic stroke, % (n)	19.70 (13)
α-Gal A activity*, Median(IQR)	0.73 (0.40,1.68)
Lyso-Gb3**, Median(IQR)	15.83 (5.09,77.90)

*α-GalA activity, α-galactosidase A activity (mmol/L/h, ref. 2.40–17.65); **Lyso-Gb3, globotriaosylsphingosine (ng/mL, ref. <1.11); hypohidrosis (reduced or absent sweating); hearing loss (including sensorineural deafness); gastrointestinal discomfort (intermittent diarrhea and constipation); FD, Fabry disease; HCM, hypertrophic cardiomyopathy.

Absolute plasma ccf-mtDNA (*MT-ND1*) and ccf-nDNA (*ACTB*) copy numbers were quantified in 37 treatment-naïve patients and 34 patients after ERT, as well as in 21 ctrls. Among the 37 treatment-naïve patients, 16 were male (13 classic, 3 non-classic) and 21 were female. A subgroup of 13 classic male patients had a median (IQR) age of 34.50 (25.25, 49.50) years and disease duration of 23.50 (10.75, 38.00) years, with key clinical features being HCM (7/13), CKD (9/13), and ischemic stroke (2/13). Baseline demographic and clinical characteristics are summarized in **Table 1**.

### 3.2 ccf-DNA levels in FD and effect of ERT

**Figure 2:**
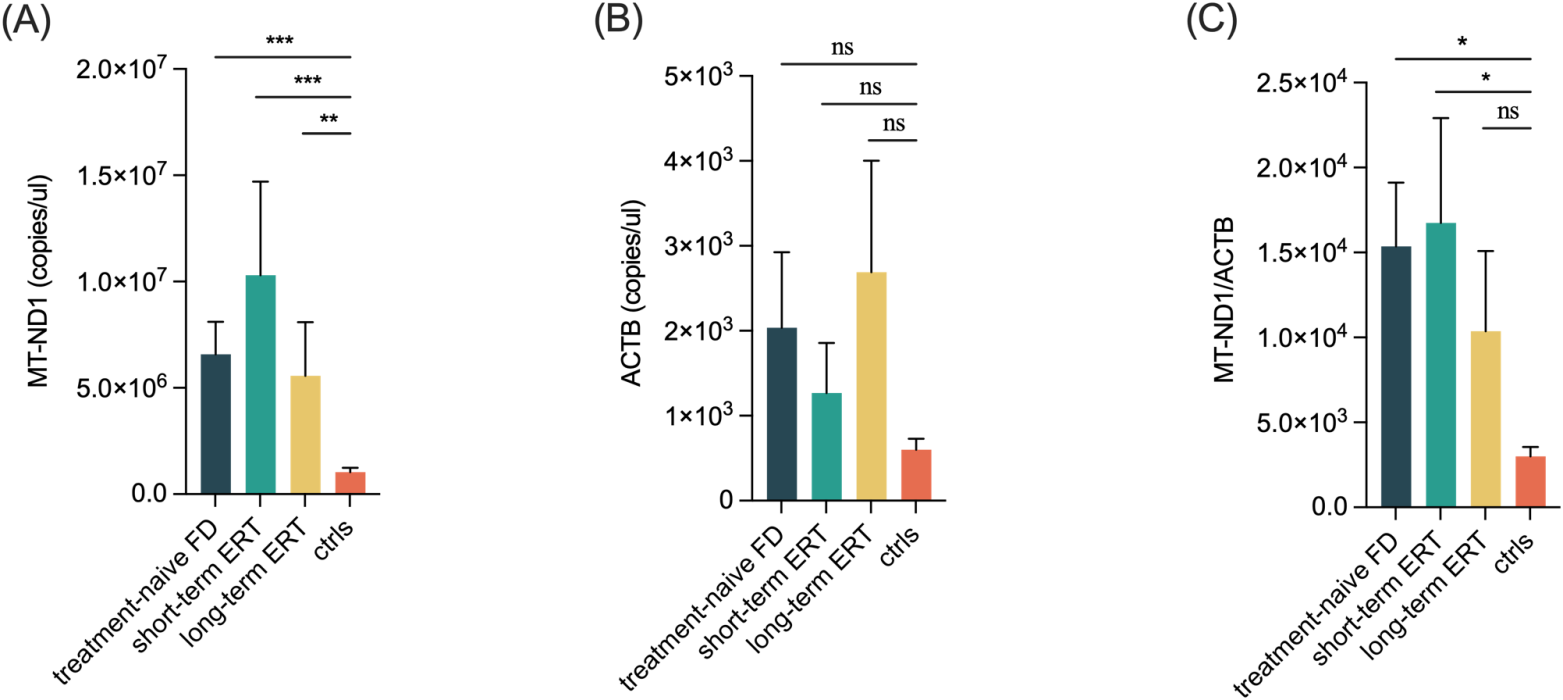
ccf-DNA levels in FD and the impact of ERT. **(A)** ccf-mtDNA, **(B)** ccf-nDNA, and **(C)** mtDNA/nDNA ratio in treatment-naïve FD, short-term ERT, long-term ERT, and ctrls groups. Data represent mean ± standard error of the mean. Ccf, circulating cell-free; FD, Fabry disease; ctrls, healthy controls; ERT, enzyme replacement therapy; *MT-ND1*, mitochondrial NADH dehydrogenase 1 gene; *ACTB*, nuclear β-ACTIN gene. ***P-adj < 0.001; **P-adj < 0.01; *P-adj < 0.05.

Both ccf-mtDNA copy number (*z*=–4.530, *P*-adj<0.001) and mtDNA/nDNA ratio (*z*=–2.613, *P*-adj=0.014) were significantly elevated in treatment-naïve patients (*n*=37) compared with ctrls (*n*=21), while no significant difference was observed in ccf-nDNA copy number (**Table 2**, **Figure 2**). In the short-term ERT group (*n*=20), both ccf-mtDNA (*z*=–4.330, *P*-adj<0.001) and mtDNA/nDNA ratio (*z*=–2.556, *P*-adj=0.017) remained elevated compared with ctrls, with no difference in ccf-nDNA. In the long-term ERT group (*n*=17), ccf-mtDNA remained elevated (*z*=–3.141, *P*-adj=0.006), whereas the mtDNA/nDNA ratio was no longer significantly different (*z*=–1.013, *P*-adj=0.311) from that of the ctrls; no differences were observed in ccf-nDNA.

**Table 2:** Ccf-DNA levels in FD and the impact of ERT.

Variables	FD	ctrls	*z*	*p*	*P*-adj
sex (male/female)	16/21	13/8			
age, Median (IQR)	34.00 (24.00,49.00)	37.00 (30.00,43.50)			
MT-ND1 (copies/µl)	3524700.64 (1462248.36,8151712.13)	817566.78 (338533.96,1396348.59)	-4.530	<0.001	<0.001***
ACTB (copies/µl)	520.18 (258.21,1032.91)	400.53 (161.02,881.28)	-1.060	0.289	0.289
MT-ND1/ACTB	6413.72 (1591.73,20022.01)	2427.42 (727.09,4788.93)	-2.613	0.009	0.014*
Variables	short-term ERT group	ctrls	*z*	*p*	*P*-adj
sex (male/female)	11/9	13/8			
age, Median (IQR)	44.00 (32.25,59.00)	37.00 (30.00,43.50)			
MT-ND1 (copies/µl)	2948037.77 (1704789.09,7098943.67)	817566.78 (338533.96,1396348.59)	-4.330	<0.001	<0.001***
ACTB (copies/µl)	599.64 (333.39,855.49)	400.53 (161.02,881.28)	-1.278	0.201	0.201
MT-ND1/ACTB	5616.19 (1977.07,21889.79)	2427.42 (727.09,4788.93)	-2.556	0.011	0.017*
Variables	long-term ERT group	ctrls	*z*	*p*	*P*-adj
sex (male/female)	13/4	13/8			
age, Median (IQR)	39.00 (34.50,50.00)	37.00 (30.00,43.50)			
MT-ND1 (copies/µl)	2137141.3 (1105795.92,4656072.06)	817566.78 (338533.96,1396348.59)	-3.141	0.002	0.006**
ACTB (copies/µl)	562.61 (319.71,1926.1)	400.53 (161.02,881.28)	-1.351	0.177	0.2655
MT-ND1/ACTB	3424.52 (1220.07,10099.08)	2427.42 (727.09,4788.93)	-1.013	0.311	0.311

*P-adj < 0.05; **P-adj < 0.01; ***P-adj < 0.001. Ccf, circulating cell-free; FD, Fabry disease; ctrls, healthy controls; ERT, enzyme replacement therapy; *MT-ND1*, mitochondrial NADH dehydrogenase 1; *ACTB*, nuclear β-ACTIN.

Furthermore, we compared ccf-DNA levels between short-term and long-term ERT groups. No significant differences in ccf-mtDNA, ccf-nDNA or the mtDNA/nDNA ratio were observed between the two groups (**Table 3**).

**Table 3:** Ccf-DNA levels between short-term and long-term ERT groups with FD.

Variables	Short-term ERT group	Long-term ERT group	*z*	*p*
sex (male/female)	11/9	13/4		
age, Median (IQR)	44.00 (32.25,59.00)	38.00 (34.50,50.00)		
MT-ND1 (copies/µl)	2948037.77 (1704789.09,7098943.67)	2137141.3 (1105795.92,4656072.06)	-1.219	0.223
ACTB (copies/µl)	599.64 (333.39,855.49)	562.61 (319.71,1926.1)	-0.213	0.831
MT-ND1/ACTB	5616.19 (1977.07,21889.79)	3424.52 (1220.07,10099.08)	-1.219	0.223

Ccf, circulating cell-free; FD, Fabry disease; ERT, enzyme replacement therapy; MT-ND1, mitochondrial NADH dehydrogenase 1; ACTB, nuclear β-ACTIN.

### 3.3 Diagnostic performance of ccf-DNA

**Figure 3:**
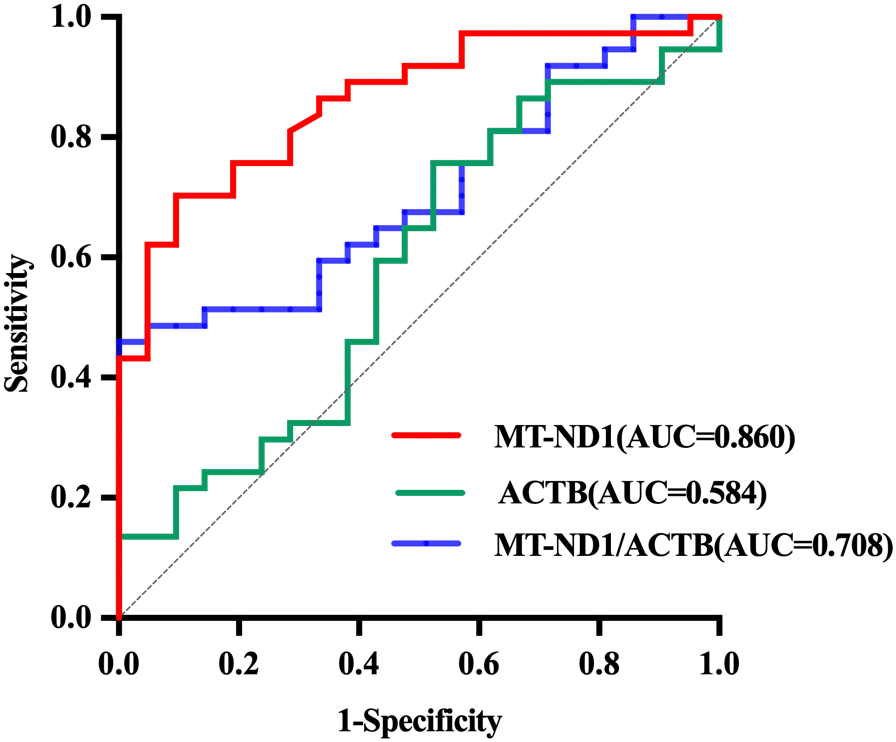
Receiver operating characteristic curves showing diagnostic performance of ccf-mtDNA, ccf-nDNA, and the mtDNA/nDNA ratio in distinguishing patients with FD from ctrls. Area under the curve (AUC) values were 0.860, 0.584, and 0.708, respectively.

Having shown the levels of ccf-DNA in FD and the impact of ERT, we next validated the discriminative potential of ccf-mtDNA for FD using receiver operating characteristic curve analysis (**Figure 3**). Ccf-mtDNA demonstrated moderate discriminative ability between patients with FD and ctrls (AUC: 0.860; 95% confidence interval: 0.766–0.955; *p*<0.001). The sensitivity of ccf-mtDNA for detecting FD was 70% with a specificity of 91%; the optimal cut-off value was 1,793,188.04 copies.

In contrast, ccf-nDNA had limited diagnostic value, with a cut-off of 294.51 copies yielding 76% sensitivity and 48% specificity (AUC: 0.584; 95% confidence interval: 0.428–0.740; *p* =0.289). The mtDNA/nDNA ratio provided high specificity but low sensitivity, with a cut-off of 8,774.04 copies achieving 46% sensitivity and 100% specificity (AUC: 0.708; 95% confidence interval: 0.576–0.839; *p*=0.009).

### 3.4 Associations of ccf-DNA with inflammatory cytokines

**Figure 4:**
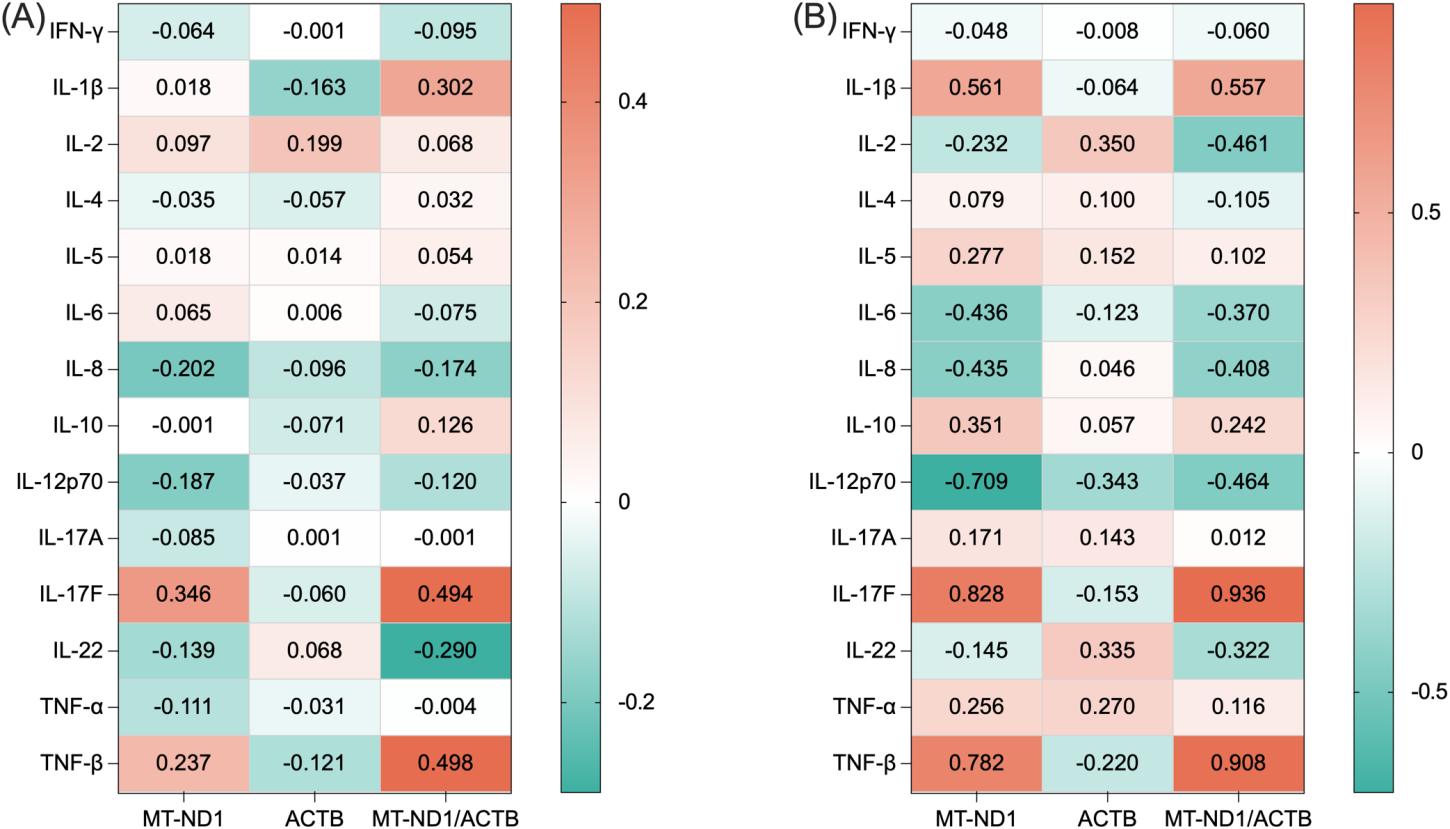
Correlations between ccf-DNA and inflammatory cytokines. **(A)** Correlations in 37 treatment-naïve patients with FD. **(B)** Correlations in 13 male patients with classic FD. “The value in the unit” represents the magnitude of the correlation between ccf-DNA and inflammatory cytokines. Positive correlations are shown in orange, negative correlations in green, and a deeper color indicates a stronger correlation. *MT-ND1*, mitochondrial NADH dehydrogenase 1 gene; *ACTB*, nuclear β-ACTIN gene; IFN-γ, interferon-gamma; IL, interleukin; TNF, tumor necrosis factor.

Next, to explore potential links with inflammation, we examined the associations between ccf-DNA and inflammatory cytokines. In the 37 treatment-naïve patients, mtDNA/nDNA ratio correlated positively with IL-17F (*r*=0.494, *P*-adj=0.021) and TNF-β (*r*=0.498, *P*-adj=0.028) after adjustment for age and sex. No correlations were observed between ccf-mtDNA or ccf-nDNA and inflammatory cytokines (**Figure 4A**, **Table 4**). In a subset of 13 male patients with classic FD, both ccf-mtDNA and mtDNA/nDNA ratio were significantly positively correlated with IL-17F (*r*=0.828, *P*-adj=0.013 and *r* =0.936, *P*-adj <0.001, respectively), and TNF-β (*r*=0.782, *P*-adj =0.021; *r*=0.908, *P*-adj<0.001) after adjustment for age. In contrast, ccf-mtDNA was negatively correlated with IL-12p70 (*r*=–0.709, *P*-adj =0.047). No correlations were observed between ccf-nDNA and inflammatory cytokines (**Figure 4B**, **Table 4**). In a subset of female patients with FD, no correlations were observed between ccf-DNA and inflammatory cytokines (**Table 4**).

**Table 4:** Correlations of ccf-DNA with inflammatory cytokine expression.

Variables	Statistic	Treatment-naïve FD patients (n=37)			Classic male patients (n=13)			Female patients (n=21)		
		MT-ND1 (copies/µl)	ACTB (copies/µl)	MT-ND1/ACTB	MT-ND1 (copies/µl)	ACTB (copies/µl)	MT-ND1/ACTB	MT-ND1 (copies/µl)	ACTB (copies/µl)	MT-ND1/ACTB
IFN-γ	*r*	-0.064	-0.001	-0.095	-0.048	-0.008	-0.06	-0.102	0.084	-0.087
	*P*	0.715	0.993	0.589	0.883	0.979	0.854	0.67	0.724	0.714
	P-adj	1.001	1.069	1.031	0.883	0.979	0.92	0.853	1.267	1.25
IL-1β	*r*	0.018	-0.163	0.302	0.561	-0.064	0.557	-0.176	-0.207	0.183
	*P*	0.919	0.35	0.078	0.058	0.843	0.06	0.457	0.382	0.441
	P-adj	0.99	2.45	0.364	0.203	1.073	0.28	1.066	5.348	2.058
IL-2	*r*	0.097	0.199	0.068	-0.232	0.35	-0.461	0.156	-0.141	0.464
	*P*	0.58	0.251	0.696	0.467	0.265	0.131	0.511	0.552	0.039
	P-adj	1.16	3.514	0.974	0.654	3.71	0.367	1.022	1.546	0.546
IL-4	*r*	-0.035	-0.057	0.032	0.079	0.1	-0.105	-0.007	-0.145	0.132
	*P*	0.844	0.744	0.856	0.807	0.758	0.746	0.976	0.542	0.579
	P-adj	1.074	1.302	0.999	0.869	1.061	0.949	0.976	1.897	2.027
IL-5	*r*	0.018	0.014	0.054	0.277	0.152	0.102	-0.178	-0.009	0.049
	*P*	0.917	0.934	0.756	0.384	0.637	0.751	0.453	0.971	0.837
	P-adj	1.07	1.189	0.962	0.672	1.274	0.876	1.268	0.971	1.172
IL-6	*r*	0.065	0.006	-0.075	-0.436	-0.123	-0.37	0.426	-0.024	0.117
	*P*	0.709	0.972	0.668	0.157	0.703	0.237	0.061	0.92	0.623
	P-adj	1.103	1.134	1.039	0.44	1.094	0.474	0.854	1.073	1.454
IL-8	*r*	-0.202	-0.096	-0.174	-0.435	0.046	-0.408	0.124	-0.079	0.045
	*P*	0.244	0.582	0.316	0.158	0.887	0.188	0.603	0.742	0.849
	P-adj	1.139	2.037	0.885	0.369	0.955	0.439	0.844	1.154	1.081
IL-10	*r*	-0.001	-0.071	0.126	0.351	0.057	0.242	-0.098	0.179	0.065
	*P*	0.993	0.684	0.472	0.264	0.862	0.449	0.683	0.449	0.784
	P-adj	0.993	1.915	1.101	0.528	1.006	0.698	0.797	3.143	1.22
IL-12p70	*r*	-0.187	-0.037	-0.12	-0.709	-0.343	-0.464	-0.152	0.1	-0.011
	*P*	0.281	0.834	0.493	0.01	0.276	0.129	0.523	0.675	0.963
	P-adj	0.984	1.297	0.986	0.047*	1.932	0.452	0.814	1.35	1.037
IL-17A	*r*	-0.085	0.001	-0.001	0.171	0.143	0.012	-0.253	0.158	-0.002
	*P*	0.627	0.997	0.994	0.595	0.659	0.972	0.282	0.505	0.994
	P-adj	1.097	0.997	0.994	0.757	1.153	0.972	1.316	2.357	0.994
IL-17F	*r*	0.346	-0.06	0.494	0.828	-0.153	0.936	-0.045	0.012	0.104
	*P*	0.042	0.731	0.003	<0.001	0.636	<0.001	0.851	0.959	0.663
	P-adj	0.588	1.462	0.021*	0.013*	1.484	<0.001***	0.916	1.033	1.326
IL-22	*r*	-0.139	0.068	-0.29	-0.145	0.335	-0.322	-0.194	0.121	-0.292
	*P*	0.426	0.698	0.091	0.653	0.287	0.307	0.412	0.612	0.211
	P-adj	1.193	1.629	0.319	0.762	1.339	0.537	1.442	1.428	1.477
TNF-α	*r*	-0.111	-0.031	-0.004	0.256	0.27	0.116	-0.3	-0.053	-0.031
	*P*	0.524	0.859	0.982	0.422	0.395	0.72	0.199	0.826	0.897
	P-adj	1.223	1.203	1.058	0.656	1.383	1.008	1.393	1.156	1.047
TNF-β	*r*	0.237	-0.121	0.498	0.782	-0.22	0.908	-0.155	-0.051	0.128
	*P*	0.17	0.49	0.002	0.003	0.491	<0.001	0.514	0.832	0.59
	P-adj	1.19	2.287	0.028*	0.021*	1.375	<0.001***	0.9	1.059	1.652

***P<0.001; * P-adj <0.05. MT-ND1, mitochondrial NADH dehydrogenase 1 gene; ACTB, nuclear β-ACTIN gene; IFN: interferon; IL: interleukin; TNF: tumor necrosis factor.

### 3.5 Associations of ccf-DNA with clinical phenotype

Finally, to evaluate the clinical value of ccf-DNA, we evaluated its relationships with clinical parameters. In the 37 treatment-naïve patients, no correlations were observed with disease duration, α-GalA activity, or plasma Lyso-Gb3 in the overall cohort after adjustment for age and sex. No correlations were observed with α-GalA activity or plasma Lyso-Gb3 in the subset of 13 male patients with classic FD after adjustment for age (**Supplementary 1**).

In the 37 treatment-naïve patients, ccf-mtDNA, ccf-nDNA, and mtDNA/nDNA ratio showed no significant correlations with clinical markers, including MSSI, LVMI, eGFR, ACR, PCR, 24h-P, Fazekas score, or VAS score, after adjustment for age and sex (**Supplementary 1**). No significant differences in ccf-DNA were found across subgroups defined by sex, *GLA* mutation type (truncated *vs*. non-truncated), or FD subtype (classic *vs*. non-classic). Similarly, no significant differences in ccf-DNA levels were detected between patients stratified by disease severity or organ involvement (high *vs*. low MSSI, HCM *vs*. non-HCM, CKD *vs*. non-CKD, mild *vs*. severe white matter lesions, neuralgia *vs*. non-neuralgia, or mild *vs*. severe pain) (**Supplementary 2**).

## 4 Discussion

The key finding of this study is the demonstration of serological evidence of mitochondrial damage in FD. We identified elevated plasma ccf-mtDNA and an increased mtDNA/nDNA ratio in patients with FD, with the high AUC for ccf-mtDNA supporting its value as a diagnostic biomarker. While FD has classically been attributed to the accumulation of Gb3 and its deacylated form, Lyso-Gb3, growing evidence indicates that additional mechanisms contribute to disease progression, including oxidative stress, impaired energy metabolism, defective autophagy, and disturbed intracellular trafficking (12). Mitochondrial dysfunction, independent of Gb3 accumulation, is emerging as a central contributor to FD pathology. Elsaid et al. (24) reported that renal tissues from *gla^–/–^* mutant zebrafish exhibited downregulation of lysosomal- and mitochondrial-related proteins, altered glycolysis and galactose metabolism, and mitochondrial structural abnormalities. Additionally, real-world outcome studies have demonstrated that long-term ERT does not prevent the progression of cardiac, renal, or cerebrovascular complications (4, 25, 26), and its benefits remain limited in paediatric patients (26).

It is generally accepted that Lyso-Gb3 is a good biomarker, and is regularly monitored during ERT. Hughes et al. (18) observed a reduction in plasma lyso-Gb3 concentration from baseline over time, with the most drastic reductions occurring in the first year of treatment. In the current study, we selected 12 months as the borderline to observe ccf-DNA change trends after ERT. Whether compared with ctrls or with short-term ERT, ccf-mtDNA levels in long-term ERT remained elevated, while ccf-nDNA levels were unchanged; additionally, the mtDNA/nDNA ratio normalized only in the long-term ERT group compared with ctrls. These findings may suggest that prolonged ERT could influence the pattern of cellular injury, possibly through improving mitochondrial integrity or reducing the relative contribution of mitochondrial damage, shifting the burden toward nuclear sources.

We also identified associations between mitochondrial damage and a subset of inflammatory cytokines, suggesting that mitochondrial dysfunction may drive inflammatory activation in FD. In lysosomal storage diseases, impaired mitophagy results in the accumulation of dysfunctional mitochondria; these release mtDNA into the cytosol and activate innate immune signaling through the cGAS–STING pathway (27–31). Once triggered, this cascade induces IFN-β and IF-stimulated gene expression (32) and promotes the release of multiple cytokines, including type I IFN, TNF-α, IL-1β, IL-18, and IL-6 (33, 34). In addition, extracellular ccf-mtDNA can further amplify inflammation through Toll-like receptor 9 signaling; mtDNA fragments activate this receptor on endo-lysosomal membranes, stimulating nuclear factor-κB and driving transcription of pro-inflammatory cytokines, including IFNs and IL-1β (35). In our cohort, ccf-mtDNA negatively correlated with IL-12p70 in male patients with classic FD. This may reflect a bimodal immune response, as reported by Posseme et al. (36), raising a possibility that IL-12p70 exerts protective pro-inflammatory effects in FD that warrant further investigation.

Although ccf-mtDNA demonstrated high specificity, its limited sensitivity and the absence of a clear association with disease severity suggest that it may function as a supportive diagnostic biomarker rather than a reliable indicator for disease monitoring or prognosis. Even so, these alterations remain informative for understanding the molecular pathogenesis of FD. A multicenter study (2) characterized males with classical FD as having lower eGFR, higher LVMI, and higher plasma Gb3 concentrations compared to males with non-classical FD or females with either phenotype (*p*<0.001). Before ERT, males with classical FD had a history of more events than males with non-classical disease or females with either phenotype. We analyzed the relationship between ccf-DNA and clinical markers in all treatment-naïve patients with FD. Ccf-mtDNA did not directly correlate with FD organ involvement, indicating that mitochondrial damage is not itself a primary driver of FD pathology. Unlike mitochondrial disorders, where intrinsic defects directly impair cellular function, ccf-mtDNA may instead act as an upstream trigger of immune signaling to initiate an amplification cascade, leading to robust inflammatory activation that ultimately contributes to target organ injury. The accumulation of Gb3 in lysosomes disrupts autophagy both *in vitro* and *in vivo (*37–39), impeding mitophagy (27). Leaked mtDNA subsequently activates the cGAS–STING pathway, inducing pro-inflammatory cytokine release. These cytokines, produced secondary to mitochondrial injury, may act as direct mediators of damage to vulnerable organs such as the heart and kidneys, consistent with our prior observations (11).

A key methodological consideration in this study relates to the approach used for ccf-DNA analysis. Two-step centrifugation and capillary electrophoresis-based quantification (such as the Bioanalyzer) currently represent best practice for achieving maximal plasma purity and unbiased ccf-DNA profiling (40, 41). In our protocol, plasma samples were processed promptly after collection and underwent repeated centrifugation to prevent cell lysis and reduce genomic DNA contamination, adhering to the core principle of sequential debris removal. For quantification, we employed target-specific RT-qPCR quantification, which differs from capillary electrophoresis-based fragment sizing and quantification. We acknowledge that these advanced techniques are essential for unbiased exploratory studies. However, RT-qPCR was selected for its high specificity in detecting short, locus-specific mitochondrial and nuclear fragments, which were the central analytes of interest in this study. Absolute quantification using standard curves enabled sensitive measurement of amplifiable copies of the target genes. Importantly, qPCR-based quantification of ccf-mtDNA is well established and has been successfully employed in prior studies, including that of Pyle et al. (23), supporting the suitability of this method for our research aims. Additionally, maintaining methodological continuity with our laboratory’s established workflows for other neurological and neuromuscular diseases (including amyotrophic lateral sclerosis, immune-mediated necrotizing myopathy, and mitochondrial disorders (8–10)) was a deliberate choice, as such consistency facilitates future cross-disease comparisons within our research program. While advanced techniques would offer additional resolution for exploratory analyses, our protocol was appropriate for the specific analytical goals of this study, and future work incorporating these state-of-the-art approaches may further extend our findings.

### 4.1 Limitations

This study had several limitations. As this was an exploratory study and no prior data on ccf-DNA in FD were available to guide sample size estimation, a formal sample size calculation was not performed; all eligible cases from our center were included. The sample size was also relatively small, particularly for subgroup analyses, which may reduce statistical power and limit the translation of our findings to different clinical settings. Additionally, although our data suggests a link between mtDNA release and inflammatory activation, this causal pathway has not been fully established and requires further investigation. Functional validation of the underlying mechanisms, such as cGAS–STING activation or impaired mitophagy, was not performed and should be addressed in future studies.

### 4.2 Conclusions

This study provides serological evidence of mitochondrial damage and its association with inflammatory activation in FD. Our findings support ccf-mtDNA as a valuable diagnostic biomarker for FD. Although no strong correlations were observed between ccf-mtDNA and clinical markers of disease burden, our results, together with our previous findings (11), indicate that targeting mtDNA-induced inflammatory pathways may provide additional therapeutic benefit. Such approaches may be particularly relevant for patients with high inflammatory activity, suboptimal ERT response, or poor prognosis.

## Data Availability

The original contributions presented in the study are included in the article/**Supplementary Material**. Further inquiries can be directed to the corresponding author.
